# Nurse employment contracts in Chinese hospitals: impact of inequitable benefit structures on nurse and patient satisfaction

**DOI:** 10.1186/1478-4491-12-1

**Published:** 2014-01-13

**Authors:** Jingjing Shang, Liming You, Chenjuan Ma, Danielle Altares, Douglas M Sloane, Linda H Aiken

**Affiliations:** 1 School of Nursing, Columbia University, 617 West 168th Street, New York, NY 10032, USA; 2 School of Nursing, Sun Yat-sen University, 74 Zhongshan 2 Road, Guangzhou 510089, China; 3 The National Database of Nursing Quality Indicators, University of Kansas, 3901 Rainbow Blvd, Kansas City, KS 66160, USA; 4 School of Nursing, University of Pennsylvania, 420 Guardian Drive, Philadelphia, PA 19104, USA

**Keywords:** Contract-based nurses, ‘bianzhi’ nurses, Nurse dissatisfaction, Patient satisfaction, Chinese hospitals

## Abstract

**Purpose:**

Ongoing economic and health system reforms in China have transformed nurse employment in Chinese hospitals. Employment of ‘bianzhi’ nurses, a type of position with state-guaranteed lifetime employment that has been customary since 1949, is decreasing while there is an increase in the contract-based nurse employment with limited job security and reduced benefits. The consequences of inequities between the two types of nurses in terms of wages and job-related benefits are unknown. This study examined current rates of contract-based nurse employment and the effects of the new nurse contract employment strategy on nurse and patient outcomes in Chinese hospitals.

**Methods:**

This cross-sectional study used geographically representative survey data collected from 2008 to 2010 from 181 hospitals in six provinces, two municipalities, and one autonomous region in China. Logistic regression models were used to estimate the association between contract-based nurse utilization, dissatisfaction among contract-based nurses, nurse intentions to leave their positions, and patient satisfaction, controlling for nurse, patient, and hospital characteristics.

**Principal Results:**

Hospital-level utilization of contract-based nurses varies greatly from 0 to 91%, with an average of 51%. Contract-based nurses were significantly more dissatisfied with their remuneration and benefits than ‘bianzhi’ nurses who have more job security (*P* <0.01). Contract-based nurses who were dissatisfied with their salary and benefits were more likely to intend to leave their current positions (*P* <0.01). Hospitals with high levels of dissatisfaction with salary and benefits among contract-based nurses were rated lower and less likely to be recommended by patients (*P* < 0.05).

**Conclusions:**

Our results suggest a high utilization of contract-based nurses in Chinese hospitals, and that the inequities in benefits between contract-based nurses and ‘bianzhi’ nurses may adversely affect both nurse and patient satisfaction in hospitals. Our study provides empirical support for the ‘equal pay for equal work’ policy emphasized by the China Ministry of Health’s recent regulations, and calls for efforts in Chinese hospitals to eliminate the disparities between ‘bianzhi’ and contract-based nurses.

## Background

National economic reform since 1978 and ongoing health system reforms [1] in China have transformed the Chinese hospital employment system. As a result, Chinese hospitals have been gradually reducing the number of nurses with ‘iron rice bowl’ positions with life-time job security, also called ‘bianzhi’ nurses, and increasing employment of contract-based nurses who have less job security and fewer employment benefits. This employment transition has led to potential inequities in Chinese hospitals, one of which is the differential treatment of contract-based nurses as opposed to ‘bianzhi’ nurses, two categories of professional nurses with the same patient care responsibilities. Reportedly 20 to 54% of nurses in Chinese hospitals have contract-based employment [2-4], and this number is expected to increase as China continues its transition from a centrally planned economy to a free market economy and demand for health care continues to increase due to health care reforms [1] and an aging population in China. However, little is known about contract-based nurses and the impact of the potential inequities of payments and benefits between contract-based nurses and ‘bianzhi’ nurses on nurse and patient outcomes.

### Chinese employment system

Since the establishment of the People’s Republic of China in 1949 and until the beginning of economic reforms in 1978, China maintained a centrally planned economy and adopted a special employment system in which the employment decisions were made by the Chinese government, and employers had little control over employment decisions and employee salary and benefits [5]. Under this system, the government decided the number of personnel for each employer, which was called ‘bianzhi’ (编制) [6]. Positions with the designation of “bianzhi’ came with an expectation of lifetime employment, steady income, and extensive benefits including housing, health insurance, and pension. A position with ‘bianzhi’ designation was guaranteed by the government and could not be dismissed by an individual employer. The ‘bianzhi’ jobs were considered by the Chinese public as formal positions and were also referred to as ‘iron rice bowl’ jobs because of their guarantee of continuing wages and benefits [5]. In addition to ‘bianzhi’ jobs, there were jobs outside ‘bianzhi’ , which were used when the allocated ‘bianzhi’ jobs could not meet employers’ operation needs. These positions outside ‘bianzhi’ were referred to as contract-based jobs (合同制) or ‘bianwai’ (编外) jobs [6]. Unlike the ‘bianzhi’ job, conditions of employment of a contract-based position are made by an individual employer, presumably based on current needs and resources. Contract-based workers do not hold lifetime employment and job-related benefits vary between employers. From 1949 through 1978, the majority of Chinese jobs were within ‘bianzhi’ or ‘iron rice bowl’ jobs [6]. However, since the 1978 reforms, China has been steadily moving from a centrally planned economy to a market economy. Consequently the Chinese employment system has undergone a significant transition. One of the main changes is the decrease of ‘bianzhi’ positions and the increase of contract-based workers. Because of the job security and steady income, ‘bianzhi’ jobs are still highly sought by the Chinese public [6].

### Chinese nursing employment trends

Like other Chinese jobs, almost all professional nurse jobs were categorized as ‘bianzhi’ jobs between 1949 and 1978. In the late 1980’s, hospitals in some coastal cities started to hire contract-based nurses to relieve the temporary nursing shortage [7]. Over the past two decades, and especially in the last 5 to10 years, Chinese hospitals have faced a substantial nurse shortage [8]. In 2006, the Ministry of Health of the People’s Republic of China stated that millions more nurses were needed to meet the increasing health care needs of the Chinese people [9]. This shortage, along with the continuing transformation of the Chinese economic system, led to the evolution of two types of employment arrangements for nurses including some traditional ‘bianzhi’ jobs and an increasing proportion of contract-based nurses [7]. The proportion of contract-based nurses employed by Chinese hospitals has been reported to vary by hospitals from 20 to 54% [2-4].

Nurses who were hired with ‘bianzhi’, mostly from 5 to 10 years ago, maintain their ‘bianzhi’ nurse status. Although there is no explicit government policy regulating the proportions of ‘bianzhi’ nurses and contract-based nurses in Chinese hospitals, anecdotal information suggests that the majority of new nurses are hired as contract-based nurses and the ‘bianzhi’ nurse positions are usually reserved for those with better qualifications such as those with Bachelor of Science in Nursing (BSN) degrees or higher [10]. There is usually no difference in the descriptions of job responsibilities between contract-based nurses and ‘bianzhi’ nurses, but differences in remuneration exist [7]. As previously discussed, a ‘bianzhi’ position represents lifetime employment which is guaranteed by the state and comes with extensive benefits. Contract-based nurses are hired by hospitals and often receive lower wages, and no or reduced benefits, at the discretion of employing hospitals [7]. Therefore, ‘bianzhi’ nurse positions are highly sought after.

The practice of unequal payment for equal job responsibilities has raised general concerns about equity as well as potential job dissatisfaction among contract-based nurses [7,11]. Nurse job dissatisfaction is of concern as established evidence has linked nurses’ job dissatisfaction to nurse turnover and poor quality of patient care [12,13]. Although a small number of studies have been published in China reporting job dissatisfaction among contract-based nurses [2,7,11], they are limited by small sample sizes, local scope of inquiry, and lack of control for significant covariates such as the nurse working environment which has been linked with nurse and patient outcomes by a robust body of literature [12-14].

This study addresses the gap in knowledge about the potential consequences of the unique approach of human resource management in Chinese hospitals where both ‘bianzhi’ nurses and contract-based nurses are employed for similar job descriptions but with different wages and benefits. Specifically, we determine the impact of this two-tiered employment method on nurse and patient outcomes in Chinese hospitals. The study employs survey data from a large geographically representative sample of hospitals in China to examine current rates of contract-based nurse utilization and determines the relationship between contract-based nurse employment, contract-based nurse dissatisfaction, contract-based nurse intentions to leave, and patient satisfaction in Chinese hospitals.

### Theoretical framework

**Figure 1:**
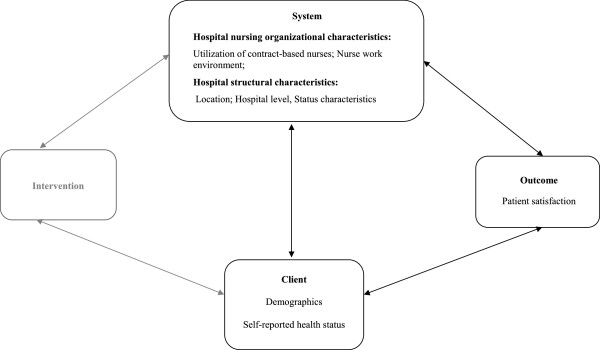
Theoretical framework adapted from the quality health outcomes model.

The quality health outcomes model by Mitchell and colleagues [15] was used to guide our analyses (Figure 1). The quality health outcomes model, derived from Donabedian’s structure-process-outcome model [15,16], includes four components: system, client, intervention, and outcome. The system component refers to the organizational characteristics of the setting where health care is provided. In our study, the system encompasses nursing organizational characteristics such as Chinese hospital two-tiered nurse employment system, nurse staffing, hospital nurse work environment, and hospital structural characteristics such as location and designated hospital level reflecting hospital resources. The client component refers to the characteristics of patients that could influence how they perceive their hospital experience including demographic information such as age, gender, and self-reported health status. The intervention refers to nursing care processes which are not directly measured in this study. The outcome in this study is patient satisfaction. According to Mitchell’s model, there are reciprocal relationships between the four components except for intervention and outcome [15]. Hospital organizational factors such as the utilization contract nurses and inequity of treatment between contract nurses and ‘bianzhi’ nurses affect contract nurses’ intention to leave their current position, nurse satisfaction, and ultimately patient satisfaction. On the other hand, intervention also affects system. For example, in order to optimize patient outcomes, organizations (for example hospitals) may need to undertake some changes (such as hiring more nurses with specialty, or setting up special functional teams) when adapting or implementing a new nursing intervention. Mitchell also suggested no direct relationship between interventions and outcomes, and emphasized that the direction and magnitude of the indirect effects of interventions on outcomes varies by organizations and clients [15]. More specifically, in this study the same nursing care may lead to different patient outcomes, depending upon the hospital system such as how the contract-based nurses were treated, and depending on the characteristics of the patients.

## Methods

This is a cross-sectional study using geographically representative data collected from 2008 to 2010 from 181 hospitals in six provinces, two municipalities, and one autonomous region in China. The parent project sponsored by China Medical Board (CMB) and conducted by CMB China Nursing Network was the first comprehensive study linking nursing workforce factors to quality of care in China [14].

### Sample

Hospitals were recruited from nine Chinese provinces/municipalities/autonomous regions which spanned all eight economic zones in mainland China. The survey method, which has been described in detail previously [14], involves a stratified and purposive sample that recruited 20 hospitals from each of eight geographic areas, except for one research site where 21 hospitals were recruited. Equal numbers of level 2 hospitals (300 to 500 beds) and level 3 hospitals (over 500 beds) were drawn. In addition to the differences in bed size, level 3 hospitals, unlike level 2 hospitals, are usually major hospitals with high technology capacity and resources to care for more complex patients. The hospital sample was stratified to represent different urban community contexts (municipality, capital cities, and non-capital cities) and different sponsorship (provincial hospitals, municipal hospitals, and university hospitals). The response rate (agreement to participate) at the hospital level was 96%, and the few hospitals that refused to participate among the hospitals that were initially sampled were replaced by hospitals at the same level and in the same location categories. Thus while China is a very large country, the systematic sampling of hospitals is believed to have resulted in a hospital study population reasonably representative of level 2 and level 3 hospitals that care for patients with complex medical conditions.

After hospital selection, at least four units were randomly chosen from all the medical, post-operative surgical, and ICUs in each hospital. All registered nurses from the selected units, excluding nurse managers, were informed of the purpose of the study and its voluntary nature, and were invited to participate by a designated research nurse in each hospital. Ninety-five percent of sampled registered nurses (RNs) completed the confidential surveys which were sent unopened to the research team at Sun Yat-sen University (SYSU) for analysis [14].

In each selected unit patients with at least 3-day inpatient stays were also sampled on a designated day with a minimum target of 5 patients per unit, and 30 patients from each hospital. A 3-day stay has been established by previous research to result in patient satisfaction assessments with predictive validity [17,18]. The overall response rate for the patient survey was 89%, with a total of 6,494 patients from 181 hospitals, and an average of 36 patients per hospital.

The China nurse survey was based on the well-designed and rigorously vetted University of Pennsylvania Multi-State Nursing Care and Patient Safety Study [19]. The patient survey instrument was the Agency for Healthcare Research and Quality’s (AHRQ) Hospital Consumer Assessment of Healthcare Providers and Systems (HCAHPS) survey [20]. Both survey questionnaires were translated to Mandarin and back-translated to English by two bilingual nursing researchers independently. Items that were not culturally relevant to Chinese nurses were removed or revised. Before utilization, both questionnaires were pilot tested in one Chinese hospital with high content validity [21-23].

A hospital survey was also sent to the department of nursing in each participating hospital to collect information on hospital characteristics such as teaching status, hospital level designation, number of inpatient beds, and number of medical and surgical units and ICUs. The three surveys were linked by unique hospital identification numbers prior to the data analyses.

### Measurement

In the nurse survey, each respondent reported her/his employment status as ‘bianzhi’ or contract-based nurse. The hospital-level utilization of contract-based nurses was estimated by dividing the number of surveyed contract-based nurses by the total number of surveyed nurses in each hospital.

Nurse outcomes studied included burnout, measured by the emotional exhaustion subscale of the Maslach Burnout Inventory that has been used and validated internationally [24-26]. In the analyses, we dichotomized the emotional exhaustion value by using the cutoff of 27 recommended by the instrument developer based upon scores in previous research [25], with scores higher than 27 indicating a high level of burnout. Job dissatisfaction, measured by a single-item asking nurses to rate their overall satisfaction with their current nursing job with four response categories, ranging from ‘very satisfied’ to ‘very dissatisfied’. In the survey, nurses were also asked about their satisfaction with specific features of their employment such as salary, health insurance benefits, pension, and tuition benefits. Responses to both the global and specific satisfaction questions were also dichotomized and nurses were categorized as satisfied or unsatisfied in the analyses. Nurse intent to leave was measured by a single-item asking nurses whether they plan to stay in their same job over the next year.

The nurse survey also asked nurses to assess their work environment using questions derived from the Practice Environment Scale of the Nursing Work Index (PES-NWI) [27], a measure that has been widely used [28] and endorsed by the National Quality Forum [29]. For the purpose of our analyses, individual nurse assessments of their practice environment were aggregated into a hospital-level measure, resulting in a composite score that reflected the nurse practice environment for each hospital. Nurses were also asked to report the number of patients in the units as well as the number of nurses working during their last shift. As Chinese hospitals still practice team nursing, the nurse staffing levels were calculated as the total number of patients divided by the total number of nurses and were aggregated to the hospital level.

Patient outcomes were obtained from the patient survey, an adapted version of the HCAHPS survey [20,30]. The patient outcomes that were used in this paper include two global measures of patient satisfaction. First, patients’ rating of overall hospital quality was measured using a scale ranging from 0 to 10 (with 10 indicating the best quality of care). This measure was dichotomized to contrast patients rating their hospitals 9 or 10 with patients rating their hospitals less than 9. Second, patients’ willingness to recommend the hospital to family or friends was based on a 4-point ordered measurement scale which was also dichotomized to contrast patients who would definitely recommend their hospital with patients who would not [14,17]. These two items were selected as patient outcome measures because previous literature has linked them with hospital organizational factors including nursing [14,18,31] and the measures had been pilot tested and validated in Chinese hospitals [21].

### Statistical analysis

We first described the study sample, and tested the differences between contract-based nurses and ‘bianzhi’ nurses using t-test and chi-square test. We determined the hospital level of contract-based nurse utilization, and then compared the rates among hospitals with different characteristics using F-tests (from one-way analysis of variance, ANOVA) and *t*-tests. We then used risk-adjusted logistic regression models to examine differences between ‘bianzhi’ nurses and contract-based nurses on each of the six nurse outcomes, including burnout, overall job dissatisfaction, and four specific measures of dissatisfaction including salary, health care insurance, pension, and tuition benefits. The majority (75%) of surveyed contract-based nurses reported less than five years of employment at their current hospitals, which was significantly different from the ‘bianzhi’ nurses. This finding was consistent with literature suggesting that some hospitals have a 5-year period of employment before they decide whether contract-based nurses will receive a promotion in their employment status or financially [7]. Thus, we restricted these analyses to the subgroup of nurses who had worked for their current employers for less than five years.

Finally, we employed similar logistic regressions using the entire sample to estimate associations between hospital nursing organizational factors and contract-based nurses’ intentions to leave, and to estimate the association between hospital nursing organizational factors and patient outcomes, respectively. In the analyses, hospital nursing organizational factors included: 1) the percent of contract-based nurses employed in each hospital which is treated as a continuous variable; and 2) the hospital proportion of contract-based nurses that were dissatisfied with each of the specific items: salary, health insurance benefits, pension, and tuition benefits. To facilitate interpretation of the results, we multiplied both the hospital proportion of contract-based nurses and the hospital proportion of dissatisfied contract-based nurses by 10. Therefore, each unit increase in these two measures in our models represents the effect of a 10-point increase in the percentage of contract-based nurses, or in the percentage of dissatisfied contract nurses. The effects of these two factors on the different outcomes were estimated in separate models.

All models controlled for hospital characteristics (hospital location, level, status, nurse staffing levels, and work environment), as well as nurse characteristics for the nurse outcomes and patient characteristics (including age, gender, and self-reported health status, which was used as a proxy for patient severity) for patient outcomes. Huber-White robust estimation procedures were used in all models to adjust the standard errors of the estimated parameters in order to account for the clustering of patients and nurses within hospitals. We used STATA 11.1 (StataCorp LP, College Station, TX, USA) for data analyses with the statistical significance level set at *P* < 0.05.

Ethical approval for this study was obtained from University of Pennsylvania and the data collection sites.

## Results

The final analyses include 9,698 nurses and 6,494 patients from 181 hospitals. Table 1 shows that there were 20 hospitals from each province, with the exception of Guangdong which had 21 hospitals. There were approximately equal numbers of level 2 and level 3 hospitals. About 46% of hospitals were municipal hospitals, 32% were university hospitals, and 22% were provincial hospitals. Forty (22%) hospitals were located in municipalities, 72 (40%) were in capital cities, and 69 (38%) were in non-capital cities. Contract-based nurse employment varied greatly across hospitals, ranging from 0 to 94% with an average of 51% (SD = 19%). Hospitals in Liaoning and Hebei provinces and Beijing employed significantly fewer contract-based nurses than hospitals from other areas, and university hospitals used significantly more contract-based nurses than provincial hospitals and municipal hospitals. The proportions of contract-based nurses did not differ by hospital level (level 2/level 3) or location (municipality/capital city/non-capital city) (Table 1).

**Table 1:** Differences in hospital contract-based nurse utilization rates

	**Hospital proportion of contract-based RNs**			
**Hospital characteristic**	**N (%)**	**Mean**	**SD**	***P *value**
Province/municipality/autonomous region				< 0.01
Beijing	20 (11.1)	0.40	0.19	
Liaoning	20 (11.1)	0.42	0.20	
Hebei	20 (11.1)	0.45	0.12	
Guangdong	21 (11.6)	0.53	0.19	
Xinjiang	20 (11.1)	0.53	0.16	
Hunan	20 (11.1)	0.55	0.18	
Sichuan	20 (11.1)	0.56	0.20	
Shaanxi	20 (11.1)	0.57	0.24	
Shanghai	20 (11.1)	0.57	0.16	
Hospital level				0.28
Level 3	91 (50.3)	0.53	0.20	
Level 2	90 (49.7)	0.49	0.18	
Hospital status				0.03
Provincial hospital	40 (22.4)	0.49	0.17	
Municipal hospital	82 (45.8)	0.48	0.17	
University hospital	57 (31.8)	0.56	0.22	
Location				0.22
Municipality	40 (22.1)	0.48	0.19	
Capital city	72 (39.8)	0.54	0.21	
Non-capital city	69 (38.1)	0.49	0.17	

*t*-tests and one-way ANOVA were used for the group comparisons. ANOVA, analysis of variance; RN, registered nurse; SD, standard deviation.

Table 2 shows that 4,988 of the 9,698 surveyed nurses (51%) were contract-based nurses. Compared with ‘bianzhi’ nurses, contract-based nurses were significantly younger, less likely to be married and have children, had less RN working experience, worked for their current hospital for a shorter period of time, and were less likely to have a BSN (or higher) degree. There were more male nurses among contract-based nurses than the ‘bianzhi’ nurses.

**Table 2:** Characteristics of contract-based nurses and ‘bianzhi’ nurses

	**Contract-based nurses (n = 4,988)**		**‘Bianzhi’ nurses (n = 4,710)**		
**Nurse characteristic**	**Mean**	**SD**	**Mean**	**SD**	**P value**
Age (years)	25.7	0.06	32.6	0.11	<0.01
Years of experience as a nurse	4.7	0.07	12.3	0.11	<0.01
Years of working in the current hospital	3.9	4.38	11.5	7.5	<0.01
	n	%	n	%	
Male	74	1.5	27	0.6	<0.01
Bachelor’s degree or above	743	15.0	1302	28.2	<0.01
Married	1737	34.9	3566	77.7	<0.01
Have children	1068	21.7	2843	62.7	<0.01

*t*-tests and chi-square tests were used for the group comparisons. SD, standard deviation.

Table 3 shows the effect of employment status on different nurse outcomes among nurses who worked for less than 5 years at their current hospital after controlling for individual nurse factors and the characteristics of hospitals. We found that among this subsample, contract nurse status was not associated with nurse burnout (OR = 0.94, 95% CI: 0.80 to 1.10) or overall job dissatisfaction (OR = 1.01, 95% CI: 0.86 to 1.18). However, contract-based nurses were significantly more dissatisfied with their salary (OR = 1.29, 95% CI: 1.05 to 1.59), health insurance (OR = 1.89, 95% CI: 1.55 to 2.31), pension (OR = 1.94, 95% CI: 1.50 to 2.36), and tuition benefits (OR = 1.30, 95% CI: 1.09 to 1.55) than their ‘bianzhi’ nurse colleagues. The models also revealed that hospital nurse work environment significantly (*P* < 0.01) affected all nurse outcomes (not shown in Table 3). Nurses working in hospitals with better work environment were significantly less likely to report burnout and dissatisfaction with each of the job elements (salary, health insurance benefits, and so on).

**Table 3:** Associations between nurse employment status and individual nurse outcomes among nurses with less than 5 years’ hospital tenure

**Nurse outcome**	**OR**	**95% CI**
High emotional exhaustion (n = 4,525)	0.94	0.80 to 1.10
Job dissatisfaction (n = 4,739)	1.01	0.86 to 1.18
Dissatisfaction with salary (n = 4,733)	1.29	1.05 to 1.59*
Dissatisfaction with health insurance benefit (n = 4,734)	1.89	1.55 to 2.31**
Dissatisfaction with pension benefit (n = 4,690)	1.94	1.50 to 2.36**
Dissatisfaction with tuition benefit (n = 4,663)	1.30	1.09 to 1.55**

Odds ratios and 95% confidence intervals in each row are from separate multivariate logistic regression models for each nurse outcome, and reflect the difference between contract-based nurses and ‘bianzhi’ nurses (the reference group). All models controlled for individual nurse characteristics and for characteristics of hospitals where nurses worked (including the nurse practice environment), and were adjusted for the clustering of nurses in hospitals. *P <0.05; **P <0.01. CI, confidence interval; OR, odds ratio.

Approximately 5% of all surveyed nurses expressed intention to leave their current position within a year. Contract-based nurses (5.93%) reported a significantly higher intention to leave their current position than ‘bianzhi’ nurses (4.26%) (*P* < 0.01). We found that contract-based nurses who worked in hospitals with high percentages of dissatisfied contract-based nurses had significantly higher intentions to leave, with an odds ratio ranging from 1.23 to 1.33 (Table 4). That is, every 10-point difference in the percentage of dissatisfied contract-based nurses across the four dimensions of dissatisfaction was related to increases in the odds on contract-based nurses intending to leave their current position, by amounts ranging from 23 to 33%.

**Table 4:** Associations between hospital contract-based nurse utilization, hospital contract-based nurse dissatisfactions, and contract-based nurses’ intent to leave current position (n = 4,568)

**Association**	**Contract-based nurses’ intent to leave current position within a year**		
	**OR**	**95% CI**	**P value**
Hospital proportion of contract nurses	0.92	0.82 to 1.04	0.18
Hospital proportion of contract nurses dissatisfied with salary	1.32	1.15 to 1.51***	0.000
Hospital proportion of contract nurses	0.94	0.84 to 1.06	0.31
Hospital proportion of contract nurses dissatisfied with health insurance	1.23	1.09 to 1.39**	0.001
Hospital proportion of contract nurses	0.93	0.83 to 1.05	0.22
Hospital proportion of contract nurses dissatisfied with pensions	1.24	1.10 to 1.40**	0.001
Hospital proportion of contract nurses	0.91	0.81 to 1.02	0.10
Hospital proportion of contract nurses dissatisfied with tuition benefit	1.33	1.15 to 1.55***	0.000

All models controlled for individual nurse characteristics and hospital characteristics (including the nurse practice environment), and were adjusted for the clustering of nurses in hospitals.

Odds ratios indicate the effect of a 10-point increase in the percentage of contract nurses;

odds ratios indicate the effect of a 10-point increase in the percentage of dissatisfied contract nurses.

**P < 0.01; ***P < 0.001. CI, confidence interval; OR, odds ratio.

Table 5 displays the results of logistic regression models estimating the effects of hospital utilization of contract-based nurses and each type of job dissatisfaction among contract-based nurses on two types of patient outcomes respectively. For each patient outcome, we estimated four models (1–4). Each individual model included hospital level utilization of contract-based nurses and one type of job dissatisfaction, and controlled for patient characteristics and hospital characteristics, including the nurse work environment and nurse staffing. We found no relationship between hospital contract-based nurse employment and patients’ overall ratings of their hospital or their willingness to recommend the hospital to others. However, contract-based nurses’ specific job dissatisfactions were negatively related to both measures of patient satisfaction across all models, and results were statistically significant for dissatisfaction with salary in patients’ hospital rating (Model 1A; OR = 0.95, 95% CI = 0.89 to 0.99, *P* < 0.05), and dissatisfaction with tuition benefits in whether patients would recommend their hospital (Model 4B; OR = 0.94, 95% CI = 0.89 to 0.99, *P* < 0.05).

**Table 5:** Associations between hospital utilization of contract-based nurses, contract-based nurse dissatisfaction, and patient outcomes

**Association**	**Patient-reported outcomes**			
	**Patient rating of hospital (n = 6,080)**		**Patient recommending hospital to others (n = 6,073)**	
	**OR**	**95% CI**	**OR**	**95% CI**
	**Model 1A**		**Model 1B**	
Hospital proportion of contract nurses	1.01	0.95 to 1.07	1.03	0.97 to 1.08
Hospital proportion of contract nurses dissatisfied with salary	0.95	0.89 to 0.99*	1.03	0.97 to 1.08
	**Model 2A**		**Model 2B**	
Hospital proportion of contract nurses	1.01	0.96 to 1.07	1.03	0.97 to 1.08
Hospital proportion of contract nurses dissatisfied with health insurance	0.98	0.94 to 1.03	0.96	0.92 to 1.01
	**Model 3A**		**Model 3B**	
Hospital proportion of contract nurses	1.01	0.96 to 1.07	1.03	0.97 to 1.09
Hospital proportion of contract nurses dissatisfied with pension	0.99	0.95 to 1.04	0.98	0.93 to 1.03
	**Model 4A**		**Model 4B**	
Hospital proportion of contract nurses	1.01	0.96 to 1.07	1.03	0.98 to 1.09
Hospital proportion of contract nurses dissatisfied with tuition benefit	0.97	0.92 to 1.03	0.94	0.89 to 0.99*

All models controlled for individual patient characteristics and hospital characteristics (including the nurse practice environment), and were adjusted for the clustering of patients in hospitals.

Odds ratios indicate the effect of a 10-point increase in the percentage of contract nurses;

odds ratios indicate the effect of a 10-point increase in the percentage of dissatisfied contract nurses.

*P < 0.05. CI, confidence interval; OR, odds ratio.

## Discussion

Our study is the first to describe contract-based nurse employment in Chinese hospitals on a national scale, and the first to examine its impact on patient outcomes. On average, about half of nurses in China’s more comprehensive hospitals, that is level 2 and level 3, were contract-based, and the proportion of contract-based nurses varied across hospitals from none to almost all nurses being contract-based. This variation may reflect a trend toward increasing utilization of contract-based nurses. Contract-based nurses were significantly younger, less likely to be married and have children, had less RN work experience, worked for their current hospital for a shorter period of time, less likely to have a BSN (or higher) degree, and more likely to be male.

While contract-based nurses did not differ from ‘bianzhi’ nurses in terms of job-related burnout or overall job dissatisfaction, contract-based nurses were significantly more dissatisfied with their remuneration, health insurance benefits, pensions, and tuition benefits than ‘bianzhi’ nurses. We also found that hospital level of contract-based nurse employment was not associated with nurse intent to leave or patient satisfaction. However, how dissatisfied contract-based nurses were with their wages and benefits significantly affected their intention to leave their jobs as well as patients’ satisfaction with their care.

Unlike 30 years ago when contract-based nurses were rare in Chinese hospitals, contract-based nurses are now commonplace. Nationwide, approximately half of the nurse workforce was composed of contract-based nurses in 2008, a number that is expected to increase as China continues its transition to a market economy [32]. Our findings confirm those from previous studies [2,4,11] suggesting that compensation and benefit differences between types of employment categories (that is, ‘bianzhi’ and contract-based nurses) are associated with dissatisfaction among the faster growing component of the nursing workforce, the contract-based nurses. Also, unrecognized before our study, inequities in remuneration and benefits and their associated adverse impact on contract nurses are associated with lower patient satisfaction, an important quality of care indicator. Clearly, the differentiation of nurses by employee benefits and job security has disadvantages for the workforce and for quality of care.

We found that most surveyed hospitals have a low intent to leave rate among contract-based nurses (5.93%), however, approximately 10% of the hospitals have a rate as high as 15% to 40%, which corresponds to hospitals with high dissatisfaction rates among contract-based nurses. One Chinese hospital reported approximately 30% of newly hired contract-based nurses did leave their position within a year [4]. Additionally, a high nurse turnover rate is known to have a negative impact on hospital quality of care [33].

Our finding of higher levels of dissatisfaction with salary and benefits among contract-based nurses and their higher intent to leave their jobs is a source of concern regarding the stability and adequacy of the hospital nurse workforce in the future. China is facing increasing demands for health care because of its growing population and increasing life expectancy [34]. At the same time, China is also experiencing a substantial nursing shortage due to the ‘inequities and imperfections’ among the Chinese health care system as well as nurse recruitment from developed countries [35]. In 2008, the China Ministry of Health published the *Nurse Regulations* which emphasized a need for ‘equal pay for equal work’ [36]. This regulation requests hospitals to provide equal salary and benefits to all nurses despite their employment type, and aims to eliminate the disparities among nurses in order to ensure that hospitals can attract and retain nurses to support a high quality of nursing care. An alternative human resource policy for hospital nurses in China is suggested by the principal results of the parent project sponsored by CMB and conducted by CMB China Nursing Network [14]. These results show that hospitals that employ a higher proportion of nurses with bachelor’s or higher degrees have substantially higher patient satisfaction and better overall quality of care. Thus a reward structure for nurses explicitly based upon educational qualifications is an evidence-based human resource policy that holds significant promise for improving quality of care in Chinese hospitals, and is a nursing workforce policy increasingly being implemented elsewhere in the world [37]. While we find that hospital nurses in China with bachelor’s degrees are more likely to hold the coveted ‘bianzhi’ positions, making educational qualifications explicit in the reward structure of nurses in the transition to a market economy warrants serious consideration.

### Limitations

This study has limitations. First, the cross-sectional study design limits the ability to detect causal relationships. Secondly, the outcome measures studied rely on self-reported patient satisfaction rather than objective outcomes such as mortality and complications. However, patient satisfaction has been previously identified as being associated with quality of hospital care as well as improved guideline adherence and lower in-patient mortality [31,38]. And, as is the case with virtually all observational studies of this sort, there may be variables omitted from our analyses that might bias our estimates or otherwise affect our results.

## Conclusion

This study describes the very common utilization of contract-based nurses in Chinese hospitals and potential inequitable practices in salary and benefits among this category of nurses. Reported dissatisfaction among contract-based nurses was significantly associated with higher contract-based nurse intent to leave their position and less satisfied patients suggesting a potential negative impact on quality of care. These findings provide support to the China Ministry of Health’s recent nurse regulations, which call for equitable treatment of contract-based nurses as a way to stabilize the nurse workforce and improve hospital quality of care [36]. Moreover, our findings also lend support to the conclusions of the China Medical Board’s parent study [14] that Chinese human resource policies in nursing should support the transition of the nursing work force in level 2 and level 3 hospitals to include explicit incentives for more nurses to have bachelor’s or higher degrees. Moving to a qualifications-based system for remunerating nurses would be consistent with current evidence and international trends.

## Abbreviations

AHRQ: Agency for Healthcare Research and Quality; ANOVA: Analysis of variance; BSN: Bachelor of Science in Nursing; CI: Confidence interval; CMB: China Medical Board; HCAHPS: Hospital Consumer Assessment of Healthcare Providers and Systems; OR: Odds ratio; PES-NWI: Practice Environment Scale of the Nursing Work Index; RN: Registered nurse; SD: Standard deviation; SYSU: Sun Yat-sen University.

## Competing interests

The authors declare that they have no competing interests.

## Authors’ contributions

JS made substantial contributions to conception, analysis and interpretation of data, manuscript writing and revision. LY contributed to acquisition of data, manuscript writing and revision. CM contributed to analysis and interpretation of data, manuscript writing and revision. DA contributed to manuscript writing and revision. DS contributed to manuscript revision for important intellectual content. LA contributed to conception, design, and manuscript writing and revision. All authors read and approved the final manuscript.
